# The microtubule plus-end-tracking protein TACC3 promotes persistent axon outgrowth and mediates responses to axon guidance signals during development

**DOI:** 10.1186/s13064-017-0080-7

**Published:** 2017-02-15

**Authors:** Burcu Erdogan, Garrett M. Cammarata, Eric J. Lee, Benjamin C. Pratt, Andrew F. Francl, Erin L. Rutherford, Laura Anne Lowery

**Affiliations:** Department of Biology, Boston College, Chestnut Hill, MA 02467 USA

**Keywords:** Microtubule, +TIPs, TACC3, XMAP215, *Xenopus laevis*, Neuronal development, Growth cone, Axon guidance

## Abstract

**Background:**

Formation of precise neuronal connections requires proper axon guidance. Microtubules (MTs) of the growth cone provide a critical driving force during navigation of the growing ends of axons. Pioneer MTs and their plus-end tracking proteins (+TIPs) are thought to play integrative roles during this navigation. TACC3 is a + TIP that we have previously implicated in regulating MT dynamics within axons. However, the role of TACC3 in axon guidance has not been previously explored.

**Results:**

Here, we show that TACC3 is required to promote persistent axon outgrowth and prevent spontaneous axon retractions in embryonic *Xenopus laevis* neurons. We also show that overexpressing TACC3 can counteract the depolymerizing effect of low doses of nocodazole, and that TACC3 interacts with MT polymerase XMAP215 to promote axon outgrowth. Moreover, we demonstrate that manipulation of TACC3 levels interferes with the growth cone response to the axon guidance cue Slit2 ex vivo, and that ablation of TACC3 causes pathfinding defects in axons of developing spinal neurons in vivo.

**Conclusion:**

Together, our results suggest that by mediating MT dynamics, the + TIP TACC3 is involved in axon outgrowth and pathfinding decisions of neurons during embryonic development.

## Background

Plus-end tracking proteins (+TIPs) selectively bind to the dynamic plus-ends of microtubules (MTs), which extend into the distal part of the axon and growth cone [1]. This enables + TIPs to come into close contact with the cell cortex, where guidance cue receptors reside. These receptors transduce asymmetrically-distributed guidance signals down to intracellular effectors, which then regulate MT dynamics in a spatially-restricted manner that likely plays a key role in growth cone turning events [2, 3]. Thus, +TIPs deserve attention for their potential function in regulating MT dynamics during axon guidance. One of the first + TIPs to be discovered for its role in axon guidance was CLASP [4]. Genetic studies in *Drosophila* demonstrated that *CLASP* is a downstream target of Abelson tyrosine kinase (Abl) in the Slit/Robo guidance pathway during central nervous system midline crossing [4]. Moreover, the + TIP and MT polymerase, *msps* (fly ortholog of XMAP215/ch-TOG) interacts with *CLASP* antagonistically during this guidance decision in an Abl-dependent manner [5]. In addition to its role in *Drosophila*, XMAP215 has been implicated in promoting axon outgrowth in vertebrates [6]. We have recently shown that the XMAP215-interactor, TACC3, is also a + TIP that regulates MT dynamics in vertebrate growth cones and is essential for normal axonal outgrowth [7]. However, how TACC3 specifically affects axon outgrowth and whether TACC3 plays a role during axon guidance remain to be explored.

In this study, we examine the role of TACC3 in axon outgrowth and pathfinding in vivo within the developing nervous system of *Xenopus laevis* which is a great model for studying cytoskeletal dynamics during axon outgrowth and guidance [8, 9]. Using time-lapse live imaging, we demonstrate that TACC3 is required for persistent axon outgrowth in *Xenopus laevis*, and that both the N- and C- terminal conserved domains of TACC3 are necessary for enhanced axon outgrowth. Moreover, TACC3-overexpressing growth cones can mitigate the reductive impacts of the MT-depolymerizing agent, Nocodazole, on MT dynamics parameters. We also show that TACC3 and XMAP215 can display a synergistic effect and promote axon outgrowth ex vivo. Finally, examination of whole mount *Xenopus* spinal cords shows defects in axon guidance in motor neurons when TACC3 levels are depleted, and manipulation of TACC3 levels impacts the growth cone response to the repellent guidance cue Slit2 in cultured *Xenopus* spinal neurons. Together, these investigations provide new insights into the mechanism by which TACC3 functions either alone or in combination with other + TIPs, such as XMAP215, to regulate MT dynamics during axon outgrowth and guidance.

## Methods

### *Xenopus* embryonic explants

Egg collection and culturing of *Xenopus* embryonic explants (from embryos of either sex) were performed as described [7, 10]. All experiments were approved by the Boston College Institutional Animal Care and Use Committee and were performed according to national regulatory standards.

### Constructs and RNA

**Fig. 1:**
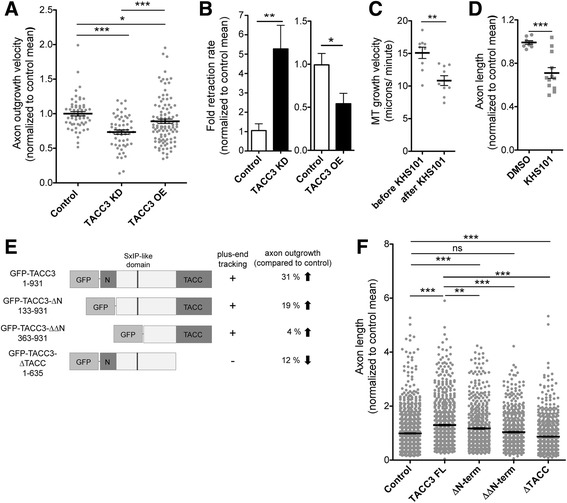
TACC3 promotes axon outgrowth velocity and prevents spontaneous axon retractions. **a**, Axon outgrowth velocity is significantly decreased in TACC3-depleted axons by 27% (*n* = 56) and in TACC3 OE, to a lesser extent, by 11% (*n* = 106) compared to control (GFP only) conditions (*n* = 58). **b,** Retraction rate increased 5 fold in TACC3 KD (*n* = 107) and decreased 0.6 fold in TACC3 OE (*n* = 155) in comparison to their corresponding non injected (*n* = 95) and GFP injected (*n* = 180) controls respectively. **c, d,** MT growth velocity (DMSO, *n* = 9, KHS-101, *n* = 9) (**c**) and axon outgrowth length (DMSO, *n* = 8, KHS-101, *n* = 12) (**d**) are significantly reduced by 28 and 26% respectively after acute depletion of TACC3 by the inhibitor KHS101. **e**, Schematic representation of GFP-tagged TACC3 full-length and deletion constructs, along with plus-end tracking ability (denoted by “+”) and impact on axon outgrowth length. **f,** Quantification of axon outgrowth length in cultured neural explants of GFP injected control (*n* = 997), full-length GFP-TACC3 (1-931aa) (*n* = 787), GFP-TACC3-ΔN (133–931) (*n* = 613), GFP-TACC3- ΔΔN (363–931) (*n* = 563) and GFP-ΔTACC domain (1-635aa) (*n* = 764). *p < 0.05, **p < 0.01, ***p < 0.001. ns not significant. *n* = axon/growth cone number

Capped mRNA constructs were transcribed and purified as previously described [6, 7] Constructs used were GFP-TACC3 (TACC3 pET30a was gift from the Richter lab, University of Massachusetts Medical School, Worcester, MA), GFP-TACC3-∆N, GFP-TACC3-∆∆N, GFP-TACC3-∆TACC (see Fig. 1e for amino acid residues for full length and each deletion construct, based on GenBank accession number NP-001081964.1) (all TACC3 constructs were subcloned into pSC2+ vector), GFP-MACF 43 (a gift from Hoogenraad Lab) in pCS2+, XMAP215-GFP (a gift from the Hyman lab, Max Planck Institute of Molecular Cell Biology and Genetics, Dresden, Germany; [11]) subcloned into pT7TS. Embryos either at the 2 cell or 4 cell stage received injections 4 times total in 0.1× MMR containing 5% Ficoll with the following total mRNA amount per embryo; 100 pg of GFP-MACF43 as a control for TACC3 or XMAP215 overexpression, 2000 pg of GFP-TACC3 full-length and deletion constructs (deletion constructs are expressed in wildtype embryos), 3000 pg of XMAP215-GFP. For double overexpression studies, 1000 pg of TACC3 and XMAP215 were injected in total.

### Morpholinos

Morpholinos (MOs) were previously described and validated [6, 7]. In knockdown (KD) experiments, TACC3 and control MOs were injected at 80 ng/embryo. For TACC3 and XMAP215 double KD analysis, 20 ng/embryo for TACC3 and control MOs and 2 ng/embryo for XMAP215 MO were injected. In rescue experiments, MO (amounts used as in KD, which is 80 ng/embryo) was injected with mRNA (same amount as in OE which is 2000 pg/embryo for GFP-TACC3 and 3000 pg/embryo for XMAP215-GFP) in the same injection solution. The efficacy of MOs has been previously assessed by Western blot of 35–36 stage embryos, as described [6, 7].

### Whole-mount immunohistochemistry

Two-day-old embryos were fixed, as described [6]. Primary antibody (diluted in blocking buffer made up by 1% DMSO, 1% Triton, 1% BSA, in PBS) to acetylated tubulin (1:1000, monoclonal, clone 6-11B-1, Sigma, St. Louis, MO, USA) and goat anti-mouse Alexa-Fluor 568 conjugate secondary antibody (1:1000, polyclonal, A-1100, Life Technologies) were used. For imaging, the spinal cord was exposed by peeling off skin, and somites were kept intact. Embryos were transferred in a drop of benzoate:benzyl alcohol (BB:BA) to the imaging chamber (made by placing Gene Frame, sticky on both sides, onto a microscope slide). After the tissue was cleared, it was covered with a 1.5× coverslip. Image acquisition and quantitation of fixed and labeled explants were described previously [6]. TACC3 KD-induced change is scored based on the percentage of embryos with disorganized axons in each condition.

### Immunocytochemistry

Embryonic explant cultures were fixed and labelled [12] with primary antibodies (1:1000 diluted in blocking buffer made up by 1% non-fat dry milk in calcium and magnesium free PBS) to tyrosinated tubulin (rat monoclonal, ab6160, Abcam) and de-tyrosinated tubulin (rabbit polyclonal, AB3201, Millipore), and with the secondary antibodies goat anti-rat Alexa Fluor 568 (1:1000, ab175476, Abcam) and goat anti-rabbit Alexa Fluor 488 (1:1000, A-11008, Life Technologies), respectively.

### Growth cone response assay

Recombinant mouse Slit2 protein (R&D Systems) (400 ng/ml) was administered to cultured neural tube explants derived from stage 28 *Xenopus* embryos in 400 μl culture media supplemented with 1% Penicillin/Streptomycin and 0.1% BSA. A perfusion chamber was set up to exchange media with Slit2-containing culture media. Time-lapse images of growth cones were acquired for 10 min with 30 s intervals before and immediately after Slit2 addition for 30 min with 30 s intervals, using a Zeiss Axio Observer inverted motorized microscope with a Zeiss 20×/0.8 Plan Apo phase objective. Frame to frame axon growth was tracked manually and retraction or growth cone collapse events were recorded over a movie. Ratio of the number of retracting frames over total frames for each axon was scored. Images given in the figures show the image of growth cone right before adding Slit2 and the image of the growth cone at collapse.

### Nocodazole application

Nocodazole to final concentration of 50 pM was administered in 400 μl culture media. Concentration of Nocodazole was determined after series of titrations and 50 pM was found to be the optimum to keep the MTs intact in order to perform MT dynamics analyses. Time-lapse images of growth cones were acquired for 1 min with 2 s intervals before and 5 min after Nocodazole administration, using a Yokogawa CSU-X1M 5000 spinning disk confocal on a Zeiss inverted motorized microscope with a Zeiss 63× Plan Apo 1.4 NA and a Hamamatsu ORCA R2 CCD camera. MT dynamics were assessed, as described [7].

### PlusTipTracker software analysis

MT dynamics were analyzed from GFP-MACF43 movies using plusTipTracker [10, 13]. Imaging conditions and tracking parameters were previously validated and same parameters were used: maximum gap length is 8 frames; minimum track length is 3 frames; search radius range 5–12 pixels; maximum forward angle, 50°, maximum backward angle, 10°; maximum shrinkage factor, 0.8; fluctuation radius, 2.5 pixels; time interval, 2 s. MT growth lifetime is the measure of persistent outgrowth till MT undergoes catastrophe. MT growth length is the total growth over a movie and MT growth velocity is the average of each MT growth event. MT dynamics parameters were compiled from multiple individual experiments and to avoid day-to-day fluctuations the final complied data were normalized to the mean of the control data for each experiment.

### Image acquisition and analysis

For axon outgrowth imaging, phase contrast images of axons were collected on a Zeiss Axio Observer inverted motorized microscope with a Zeiss 20×/0.5 Plan Apo phase objective and analyzed using ImageJ [7]. Time-lapse images for axon outgrowth velocity was collected for 4 h with 20 min intervals and images were analyzed using plusTipTracker QFSM plugin and velocity was measured as the average of instantaneous velocity per axon as described [6]. Axon retraction events were analyzed from the same data set used to assess axon growth velocity. Frame to frame axon growth was tracked manually and retraction events were recorded over a movie. Ratio of the number of retracting frames over total frames for each axon was scored.

Axon outgrowth and MT dynamics data were normalized to controls, to account for day-to-day fluctuations in room temperature. Image acquisition and quantitation of fluorescence intensity of fixed and labeled explants were described previously [7]. Experiments were performed multiple times to ensure reproducibility. Graphs were made in GraphPad Prism. Statistical differences were determined using unpaired two tailed t-tests when comparing two conditions and one-way analysis of variance with Tukey’s *post-hoc* analysis when multiple conditions were compared.

## Results

### TACC3 promotes persistent axon outgrowth by preventing spontaneous axon retractions

We previously showed that normal axonal outgrowth requires TACC3 [7]. To gain further insight into the mechanism by which TACC3 promotes axon outgrowth, we examined the effect of TACC3 knockdown (KD) and overexpression (OE) on dynamic axon outgrowth parameters. Time-lapse imaging demonstrated that TACC3 KD significantly reduced axon outgrowth velocity by 25% relative to control conditions (TACC3 KD, 0.74 ± 0.03, *n* = 46, versus control, 1.04 ± 0.03 *n* = 57, ***p < 0.0001, Fig. 1a). In addition to the reduced outgrowth velocity, TACC3 reduction dramatically increased axon retraction rates by 5-fold in comparison to control axons (TACC3 KD, 5.27 ± 1.22, *n* = 107, versus control, 1.06 ± 0.35, *n* = 99, **p = 0.0015, Fig. 1b). Conversely, when TACC3 levels were elevated, the frequency of axon retraction rates was reduced significantly by 45% compared to controls (TACC3 OE, 0.54 ± 0.12, *n* = 155, versus control, 0.99 ± 0.12, *n* = 180, *p = 0.01, Fig. 1b). Although TACC3 OE led to increased axonal length (Fig. 1f), TACC3 OE actually reduced axon outgrowth velocity by 14% (0.89 ± 0.32, *n* = 103, versus control, 1.04 ± 0.033, *n* = 57, *p < 0.0268, Fig. 1a), suggesting that the increased axonal length may result from reduced axon retraction rather than a change in outgrowth velocity.

To further explore the TACC3 KD phenotype, we examined axon outgrowth of cultured neurons in which TACC3 was acutely inhibited by the TACC3 specific inhibitor, KHS-101 [14]. Consistent with the effect seen in TACC3 KD, KHS-101-induced acute inhibition of TACC3 significantly reduced MT growth velocity by 28% (15.09 ± 0.86 μm/min (before drug treatment); 10.84 ± 0.75 μm/min (after drug), **p < 0.0019, Fig. 1c). Moreover, acute inhibition led to an immediate retraction of axon length by 26% compared to vehicle treated controls (KHS-101, 0.71 ± 0.05, *n* = 12, versus DMSO, 0.96 ± 0.03, *n* = 9, ***p = 0.0007, Fig. 1d).

In order to determine which domains of TACC3 are involved in axon outgrowth, we tested various truncation mutants of TACC3. We found that, while full-length TACC3 and ∆N (lacking conserved N-terminal domain) significantly increased axon outgrowth by 30% (1.30 ± 0.03, *n* = 787, ***p < 0.0001) and 18% (1.18 ± 0.31, *n* = 613, ***p < 0.0001) respectively, expression of ∆TACC (lacking the conserved TACC domain, which has been shown to be required for centrosome localization and interaction with the MT polymerase, XMAP215) caused a significant reduction by 12% in axon length (0.87 ± 0.021, *n* = 764, ***p = 0.0002) in comparison to wild-type neurons (0.99 ± 0.02, *n* = 997) (Fig. 1f). On the other hand, the larger N-term deletion (lacking both the conserved N-terminus and the putative SxIP-like motif that is known to mediate EB1 interaction for other + TIPs) showed no significant difference (1.04 ± 0.028, *n* = 563, *p* = 0.2012). Additionally, none of the deletion constructs that promoted axon outgrowth were as effective as full-length TACC3 OE (Fig. 1f).

Together, our findings suggest that TACC3 is required for proper axon outgrowth by opposing axonal retracting forces. Additionally, full-length TACC3 is more efficient in promoting axon outgrowth than its truncation mutants, while expression of a version lacking the TACC domain results in a mild dominant negative effect.

### TACC3 antagonizes nocodazole-induced MT depolymerization but does not affect MT lattice stability

Previously, we determined that TACC3 promotes efficient MT polymerization by enhancing MT growth velocity within growth cones [7]. However, the mechanism by which TACC3 affects MT polymerization remains to be elucidated. Thus, we sought to gain further insight by assessing the impact of low doses of the MT depolymerizing drug, nocodazole, after TACC3 manipulation.

**Fig. 2:**
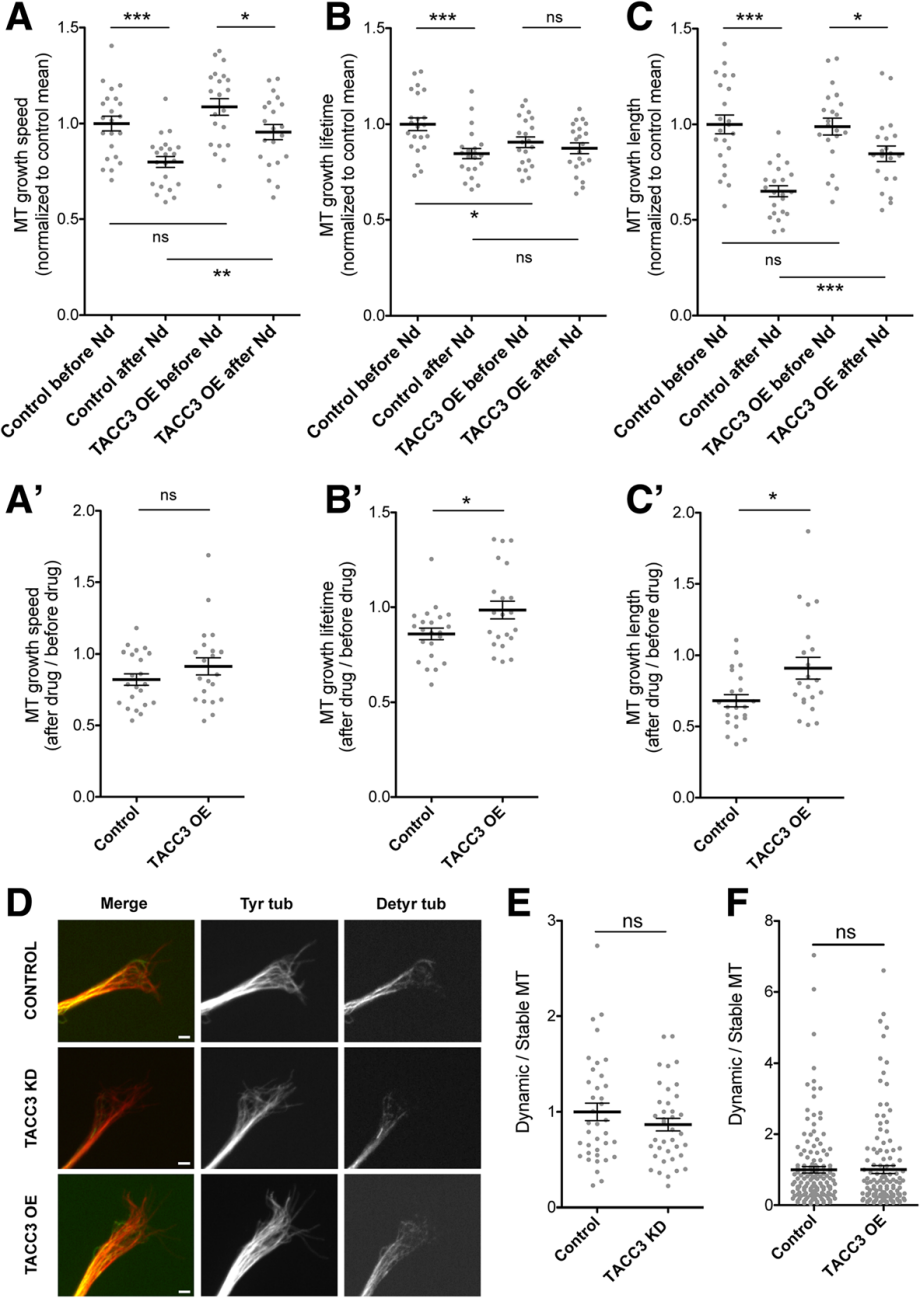
TACC3 antagonizes Nocodazole-induced MT depolymerization but does not affect MT stability. **a-c**, Quantification of the MT dynamics shows significant reduction in MT growth speed (**a**), MT lifetime (**b**) and MT growth length (**c**) in control (*n* = 22) and TACC3 OE (*n* = 21) growth cones in response to 50 pM Nocodazole before and 5 min after drug treatment. However, the effect of Nocodazole on TACC3 OE growth cones is dampened compared to controls. **a’-c’**, Although not significant, reduction in MT growth speed (**a’**) is more prominent in control growth cones compared to TACC3 OE growth cones while the reduction in both lifetime (**b’**) and length (**c’**) in control growth cones are significantly higher than the TACC3 OE growth cones (**d**) Representative growth cone images of control, TACC3 KD and TACC3 OE, immunostained for tyrosinated tubulin (*red*) and detyrosinated tubulin (*green*) to label dynamic versus stable MTs, respectively. **e-f**, Quantification of the fluorescence intensity of imaging data in G, with TACC3 KD (*n* = 38) (**e**) and TACC3 OE (*n* = 129) (**f**) growth cones showing no significant changes in dynamic/stable MTs compared to corresponding control growth cones (*n* = 37 and *n* =143, respectively). *p < 0.05, **p < 0.01, ***p < 0.001. ns not significant. *n* = growth cone number. Scale bar, 2 μm

We observed that a low dose of nocodazole led to reduction in several parameters of MT dynamics, and that TACC3 OE could mitigate these effects. While control growth cones exhibited a marked 20% decrease in MT growth speed after treatment with 50 pM nocodazole (before, 1.00 ± 0.04, *n* = 22, after treatment, 0.79 ± 0.03, *n* = 22, ***p = 0.0001), TACC3 OE growth cones showed reduction by only 12% (before, 1.08 ± 0.04, *n* = 21, after nocodazole, 0.95 ± 0.03, *n* = 21, *p = 0.0316, Fig. 2a). Similar trends were observed with MT growth lifetime, in which control growth cones showed a 15% reduction (before, 1.00 ± 0.03 s, *n* = 22, after nocodazole, 0.84 ± 0.03 s, *n* = 22,***p = 0.0008) versus only a 3% reduction with TACC3 OE (before, 0.91 ± 0.03, *n* = 21, after nocodazole, 0.87 ± 0.03, *n* = 21, *p* = 0.4305, Fig. 2b), and for MT growth length, there was a 35% reduction in controls (before, 1.00 ± 0.05, *n* = 22, after nocodazole, 0.65 ± 0.03, *n* = 22, ***p < 0.0001) versus 14% in TACC3 OE (before, 1.00 ± 0.04, *n* = 21, after nocodazole, 0.85 ± 0.04, *n* = 21, *p = 0.02, Fig. 2c). These results suggest that TACC3 can mitigate the nocodazole-induced reduction in MT growth dynamics parameters. This mitigation can be more clearly visualized when the nocodazole-induced change is represented as the ratio of after treatment/before treatment. Although the relative reduction in MT growth speed when TACC3 is overexpressed is only slightly less compared to controls and is not quite statistically significant (0.91 ± 0.06 *n* = 21 versus control 0.82 ± 0.04 *n* = 22, ns *p* = 0.2, Fig. 2a’), for other MT growth parameters, TACC3 OE significantly dampens the nocodazole-induced reduction in lifetime (0.99 ± 0.047 *n* = 21 versus control 0.86 ± 0.03 *n* = 22, *p = 0.03, Fig. 2b’) and length (0.91 ± 0.08 *n* = 21 versus control 0.68 ± 0.04 *n* = 22, *p = 0.01, Fig. 2c’) compared to controls.

In addition to MT polymerization, MT stabilization is considered to be an important parameter for axon outgrowth and growth cone turning events [12]. Hence, we measured the fluorescence intensities of tyrosinated and de-tyrosinated tubulin in the growth cone and assessed dynamic and stable MT lattice profiles, in TACC3-manipulated growth cones. We found that the ratio of tyrosinated tubulin versus de-tyrosinated tubulin did not statistically differ in TACC3 KD (0.87 ± 0.07, *n* = 37, *p* = 0.2394) nor in TACC3 OE (1.01 ± 0.11, *n* = 129, *p* = 0.9673) growth cones, with respect to control growth cones (1.00 ± 0.09, *n* = 143, Fig. 2d-f). This suggests that TACC3 may not specifically regulate MT lattice stability in growth cones.

### TACC3 and XMAP215 interact to promote axon outgrowth

**Fig. 3:**
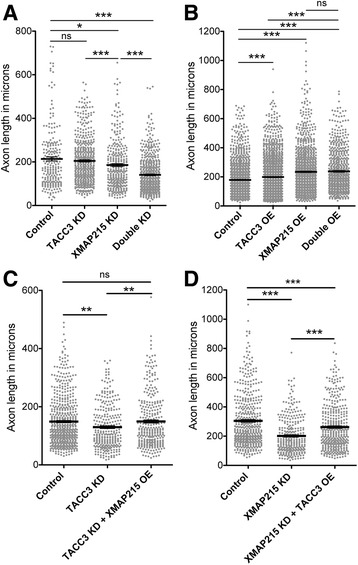
TACC3 and XMAP215 interacts to promote axon outgrowth. **a, b**, Combinatorial reduction or elevation of TACC3 and XMAP215 levels reveals synergistics in axon outgrowth. Knocking down both TACC3 and XMAP215 (*n* = 312) showed significant reduction in axon length in comparison to control (*n* = 219), TACC3 KD (*n* = 487) and XMAP21 KD alone (*n* = 312) (**a**). Overexpression of both (*n* = 654) showed significant increase in axon length in comparison to control (*n* = 1288) and TACC3 OE (*n* = 1585), while double overexpression had no additive effect in comparison to XMAP215 OE (*n* = 1227) (**b**). **c**, **d**, Reduced axon outgrowth in TACC3 KD (*n* = 289) (**c**) or XMAP215 KD (*n* = 299) (**d**) neurons is rescued by the overexpression of XMAP215 (*n* = 313) or TACC3 (*n* = 397), respectively. *p < 0.05, **p < 0.01, ***p < 0.001. ns not significant. *n* = axon number

We previously found that the TACC3 interactor and MT polymerase, XMAP215, also promotes axon outgrowth [6], and that TACC3 and XMAP215 co-localize at the extreme plus-end of MTs in growth cones in a co-dependent manner [7]. However, the consequences of their interaction on axon development have not been elucidated. Therefore, we sought to test whether TACC3 and XMAP215 might cooperate synergistically to promote axon outgrowth by partially elevating or reducing TACC3 and XMAP215 levels alone and in combination with each other. While a very mild TACC3 KD (approximately 20–30% less) led to 10% reduction in axon outgrowth (204.7 ± 4.8 μm, *n* = 487, *p* = 0.3528) and partial XMAP215 KD led to 13% reduction (185.6 ± 6.4 μm, *n* = 312, *p = 0.0116), partial knockdown of both reduced axon length significantly by 34% (140.6 ± 3.5 μm, *n* = 552, ***p < 0.0001) compared to control axons (213.6 ± 9.6 μm, *n* = 219, Fig. 3a). Conversely, overexpression of both (double OE) increased axon length by 32.7% (237.7 ± 5.4 μm, *n* = 654, ***p < 0.0001) while TACC3 OE alone increased by 11% (198.9 ± 3.2, *n* = 1585, ***p < 0.0001) and XMAP215 OE increased by 30.9% (234.5 ± 4.3 μm, *n* = 1227, ***p < 0.0001) in comparison to controls (179.1 ± 3.2 μm, *n* = 1288, Fig. 3b). Interestingly, while double OE significantly increased axon length in comparison to TACC3 OE alone (***p < 0.0001), it did not show a difference when compared to XMAP215 alone (*p* = 0.6421) suggesting that there may be an upper threshold that is reached with XMAP215 OE by itself.

We next asked whether overexpression of one + TIP might rescue the reduced axon length in the absence of the other. We observed that overexpressing XMAP215 in the stronger TACC3 KD background brought axon outgrowth length to control levels (TACC3 KD + XMAP215 OE, 149.4 ± 5.2 μm, *n* = 313, **p = 0.005, versus control, 148.8 ± 3.6 μm, *n* = 558, *p* = 0.9331) by increasing the length 15% in comparison to TACC3 KD (129.7 ± 4.477 μm, *n* = 289, **p = 0.0015, Fig. 3c). On the other hand, overexpression of TACC3 in the XMAP215 KD background increased axon outgrowth length by 30% (XMAP215 KD + TACC3 OE, 262.2 ± 7.8 μm, *n* = 397, ***p < 0.0001) in comparison to XMAP215 KD (201.3 ± 7.2 μm, *n* = 299); however, the rescue was not complete when compared to control axons (Control, 304.9 ± 8.5 μm, *n* = 463, ***p = 0.003, Fig. 3d). These findings suggest that TACC3 and XMAP215 cooperate during axon outgrowth, with XMAP215 showing more additive effects on TACC3-mediated axon outgrowth.

### TACC3 affects axon guidance in vivo and ex vivo

**Fig. 4:**
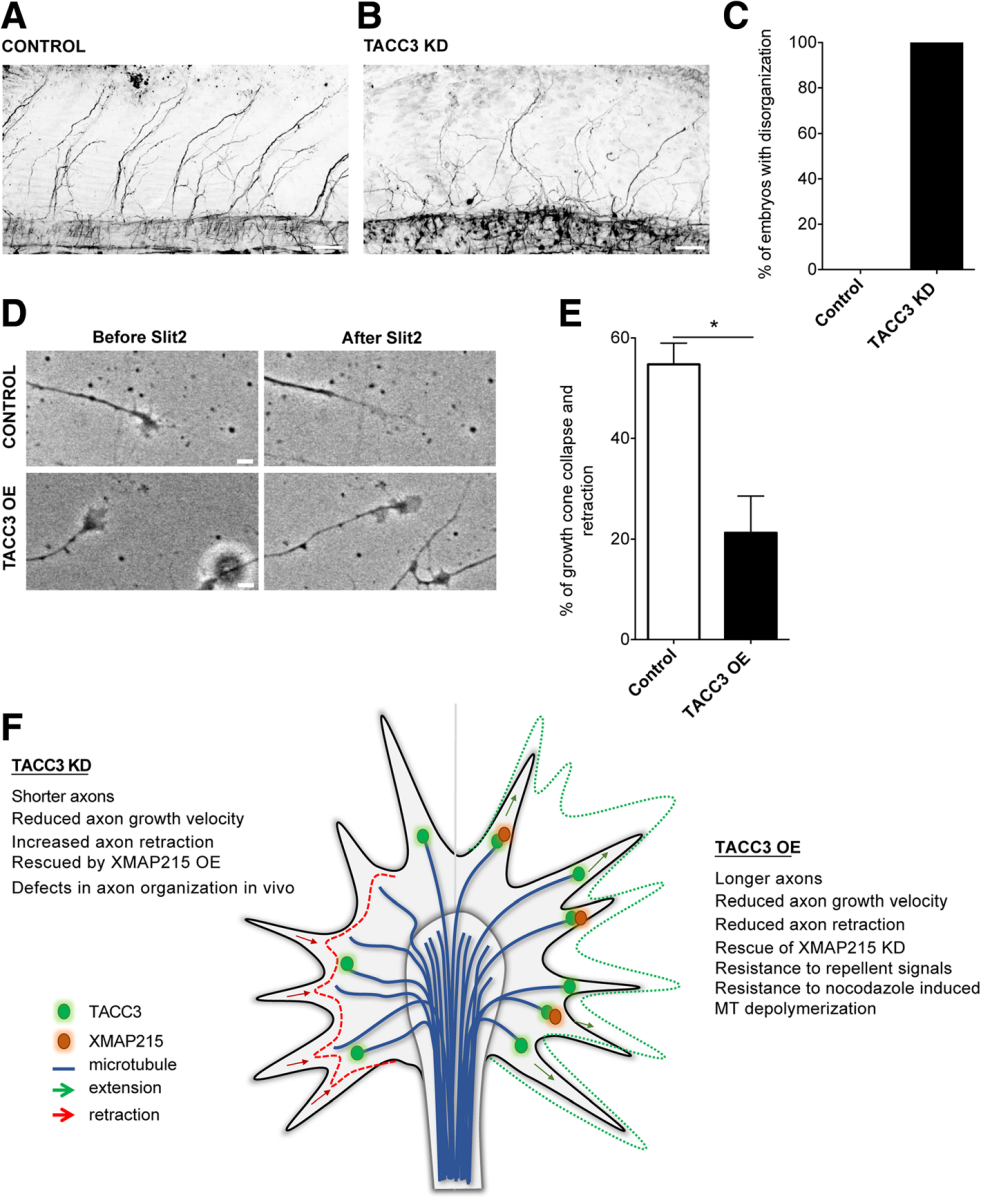
TACC3 affects axon guidance in vivo and ex vivo. **a**, **b**, confocal images of laterally-viewed whole-mount Xenopus spinal cord fluorescently labeled for acetylated tubulin, showing peripheral axon outgrowth in control (**a**) and TACC3 KD (**b**) embryos at 2 dpf. **c**, Quantitation of the embryos with motor neuron guidance defects (*n* = 5 embryos). **d**, Representative neural tube growth cone images of control and TACC3 OE, before and after addition of 400 ng/ml Slit2. **e**, Quantification of the percentage of the growth cone collapse events in control (*n* = 48) and TACC3 OE (*n* = 82) growth cones show significant reduction in growth cone collapse in TACC3 overexpressing growth cones. **f,** Cartoon model for the role of TACC3 at MT plus ends during axon outgrowth and guidance. Microtubule (*blue*) plus-ends decorated by TACC3 (*green*) promotes axon outgrowth, reduces axon retraction, dampens nocodazole induced reduction in MT dynamics parameters, rescues XMAP215 KD induced axon length reduction and opposes repellent guidance signals effect. *p < 0.05, **p < 0.01, ***p < 0.001. ns not significant. *n* = growth cone number. Scale bar, 50 μm and 5 μm

The direction that the growth cone acquires during outgrowth is a result of local modulation of MT dynamics in response to guidance signals [12, 15–17]. Thus, we wondered whether TACC3 regulation of MT dynamics could play a role during axon guidance. We first examined motor neuron axon outgrowth from the spinal cord in embryos at an early developmental stage (st 28), and we discovered that reduction of TACC3 caused significantly impaired outgrowth and severely disrupted morphology in all embryos examined (Fig. 4a-c). To gain greater insight into whether TACC3 manipulation causes this disorganization under specific guidance signals, we examined growth cone behavior in response to the guidance molecule, Slit2, applied in culture media. Slit2 is a repellent guidance cue which has been previously studied with other + TIPs, such as CLASP [4], and the response of growth cones of different neuron types isolated from *Xenopus* embryos at different stages has been previously documented [18–20]. We monitored the changes in growth cone behavior for 10 min prior and for 30 min after addition of Slit2. Growth cones that show persistent growth were picked to be analyzed for their behavior after Slit2 addition. Growth cones that had reduced lamellipodial area were considered as collapsed. We found that TACC3 OE growth cones had significantly fewer growth cone collapse and axon retraction events (TACC3 OE, 21.28 ± 7.24 *n* = 76 versus control, 54.72 ± 4.26 *n* = 44, *p = 0.0164) in response to Slit2, when compared to wild type growth cones (Fig. 4d-e). This suggests that overexpressing TACC3 can counteract Slit2-induced growth cone collapse.

## Discussion

Dynamic spatial and temporal regulation of MTs within the growth cone is considered to be of key importance during axon outgrowth, guidance decisions and regeneration events [12, 15–17, 21, 22]. Accordingly, MT plus-end tracking proteins (+TIPs) likely play a critical role during axon guidance, as + TIPs dominate the dynamic portion of MTs that reaches the growth cone periphery, where guidance cue receptors reside [2]. However, few + TIPs have been examined within the context of the embryonic growth cone. We previously characterized a MT plus-end tracking function for TACC3 and showed that it can promote MT polymerization and is required for proper axonal development [7]. Here, we sought to uncover new insights into the mechanism underlying axonal regulation by TACC3.

First, we found that the shorter axons that result from reduced levels of TACC3 were due to slower axon outgrowth velocity along with significantly increased retraction rate. Moreover, TACC3 overexpression leads to longer axons, not because of fast axon outgrowth velocity (the outgrowth rate was actually slower than in controls), but because of reduced axon retraction rate. This suggests that TACC3-mediated MT dynamics may be required for opposing the normally-occurring retractive forces within axons. Another possible explanation is that the reduced axon outgrowth velocity and reduced axonal retraction rates after TACC3 OE could be due to stronger anchorage to the underlying substrate and adhesion turnover. While there are some + TIPs that have been implicated to mediate MT – focal adhesion interactions [23], TACC3 has not yet been explored in focal adhesion (or point contacts, in the case of growth cones) regulation. However, since TACC3 has been identified as an interactor of CLASP [24], and given that CLASP is known to function during focal adhesion turnover [25], future studies should examine whether TACC3 also plays a role at focal adhesions/point contacts.

+TIPs modulate MT dynamic instability in various ways; for example, XMAP215 promotes MT growth by catalyzing addition of tubulin dimers [26], while CLASP and APC rescue MT from catastrophe by increasing MT stability [27–29]. Here, we showed that TACC3 OE can dampen nocodazole-induced reduction in MT growth speed, length and lifetime. However, this was not achieved by increased MT lattice stability, as immunofluorescence analysis of dynamic versus stable MTs revealed that TACC3 has no apparent impact on MT stability within the growth cone. It is unclear how TACC3 is able to mitigate the reduction in MT growth speed, length and lifetime as a result of nocodazole application. One possibility could be that TACC3 overexpression, which enhances XMAP215 localization at MT plus ends [7], may simply promote more efficient and processive MT polymerization by XMAP215 to counteract the nocodazole-induced effects.

Individual + TIPs comprise a network of proteins at MT plus-ends which can co-localize and function together to modulate MT dynamics. We have previously shown such cooperation between TACC3 and XMAP215 in growth cones, as we demonstrated that TACC3 and XMAP215 co-localize at MT plus-ends in co-dependent manner [7].

Here, we found that TACC3 and XMAP215 interact to promote axon outgrowth (Fig. 3). Partially knocking down both TACC3 and XMAP215 resulted in further reduction in axon outgrowth length, which suggests a synergistic interaction between the two proteins. However, overexpression of both + TIPs did not show further increase in axon length in comparison to XMAP215 OE alone. This might be due to an upper threshold that is reached with overexpression of XMAP215 alone. Conversely, rescue studies show that XMAP215 can fully restore TACC3 KD-mediated reduced axon length to control levels, whereas TACC3 OE fails to show the same impact over XMAP215 KD. As XMAP215 is a processive MT polymerase, reduction in XMAP215 levels may exert more dramatic effect than the reduction in the levels of TACC3, which may play more of an accessory role. Considering that one study suggests that every TACC3 molecule is thought to interact with two molecules of XMAP215 [30], reduced levels of XMAP215 could be a limiting factor. Even though TACC3 OE functions to increase available XMAP215 at MT plus-ends, the reduction in overall XMAP215 levels may result in poor axon outgrowth. While knock down approaches provide supporting evidence regarding the combinatorial role of TACC3 and XMAP215 during axon outgrowth, future studies should utilize mutations that disrupt their interaction [30] in order to understand the dependence of these two proteins on one another during axon outgrowth.

In addition to their role in axon outgrowth, several + TIPs have been implicated in participating in growth cone steering decisions in response to extracellular cues. The first of which is *orbit/MAST*, the fly ortholog of mammalian CLASP, that has been identified to cooperate with Abelson kinase (Abl) downstream of Slit/Robo guidance pathway [4]. In a parallel genetic and proteomic screen in fruit flies, *minispindles* (*msps*), a fly ortholog of Xenopus XMAP215, was identified to function antagonistically against CLASP and Abl during embryonic central nervous system development [5], while another genetic interaction study in flies identified *dtacc* as an antagonist of CLASP [24], reminiscent of the interaction between CLASP and TACC partner, *msps*. Combining these previous works with our findings on the role of TACC3 in axon outgrowth led us to ask whether TACC3 functions during axon guidance. As demonstrated in Fig. 4a, our initial observations revealed that reduction in TACC3 levels impairs the normal organization of axons exiting the spinal cord in embryos at st 28. Stimulation of cultured *Xenopus* retinal neurons at stage 32 or beyond with bath-applied Slit2 has been shown to cause growth cone collapse [19]. Additionally, spinal neurons derived from st 28 *Xenopus* embryos have been previously shown to be repelled by Slit2 [18]. Here, we found that Slit2-induced neural tube growth cone collapse events can be reduced by 60% in TACC3 overexpressing growth cones in comparison to control, suggesting an opposing role for TACC3 in Slit2-induced growth cone collapse. Based on its role in MT polymerization [7], its co-dependent localization at MT plus ends with XMAP215 [7], and their interaction during axon outgrowth (Fig. 3), we propose that TACC3 OE will excessively occupy MT plus-ends, subsequently driving increased recruitment of XMAP215, prompting enhanced MT polymerization in all directions. This global increase in MT polymerization would disturb local MT modulation, which is the underlying mechanism for growth cone steering events and it would result in an aberrant, non-obedient growth cone advance (Fig. 4f).

It remains to be determined whether these effects are specific to Slit2 or if TACC3 could exert similar opposing effects in response to other repellent signals, and/or if TACC3 mediates attractive signals as well. Finally, other TACC members, namely, TACC1 and TACC2, have recently been characterized as + TIPs that can promote MT polymerization in *Xenopus* embryonic cells [31, 32]. Although their expression and MT regulatory function show cell type-specificity, it would be intriguing to study whether other members of the TACC family also play a role in axon outgrowth and guidance decisions.

## Conclusion

This study characterizes the mechanism by which TACC3 regulates MT dynamics within the embryonic neuronal growth cone and promotes axon outgrowth. Using time-lapse imaging of *Xenopus laevis* embryonic axons as they grow in culture, we demonstrated that TACC3 promotes persistent axon outgrowth not by accelerating axon growth velocity but by reducing spontaneous axon retraction events. Moreover, we demonstrate that overexpressing TACC3 can mitigate the reduction in MT dynamics parameters that occur after Nocodazole application, suggesting that TACC3 may be promoting MT dynamics by dampening MT depolymerization. Finally, our data suggests that the + TIP TACC3 mediates axon guidance, as reduction in TACC3 levels results in defects in the normal organization of spinal neuron axons within the spinal cord. Moreover, bath application of the Slit2 repellent guidance molecule into cultured neural tube neurons shows that TACC3 OE reduce the Slit2 induced growth cone collapse events suggesting that TACC3 may involve in generation of response to guidance signals during neuronal development.

## Abbreviations

+TIPs: Plus-end tracking proteins
ch-TOG: Colonic and hepatic tumor overexpressed gene protein
CLASP: Cytoplasmic linker associated protein
KD: Knockdown
MO: Morpholino
msps: Minispindles
MT: Microtubule
Nd: Nocodazole
OE: Overexpression
TACC3: Transforming acidic coiled-coil
XMAP215: *Xenopus* microtubule associated protein 215
